# Analyzing Axial Stress and Deformation of Tubular for Steam Injection Process in Deviated Wells Based on the Varied (*T*, *P*) Fields

**DOI:** 10.1155/2013/565891

**Published:** 2013-09-15

**Authors:** Yunqiang Liu, Jiuping Xu, Shize Wang, Bin Qi

**Affiliations:** ^1^Uncertainty Decision-Making Laboratory, Sichuan University, Chengdu 610064, China; ^2^College of Economics & Management, Sichuan Agricultural University, Chengdu 611130, China; ^3^Research School of Engineering Technology, The Southwest Petroleum and Gas Corp, China Petroleum and Chemical Corp, Deyang 618000, China

## Abstract

The axial stress and deformation of high temperature high pressure deviated gas wells are studied. A new model is multiple nonlinear equation systems by comprehensive consideration of axial load of tubular string, internal and external fluid pressure, normal pressure between the tubular and well wall, and friction and viscous friction of fluid flowing. The varied temperature and pressure fields were researched by the coupled differential equations concerning mass, momentum, and energy equations instead of traditional methods. The axial load, the normal pressure, the friction, and four deformation lengths of tubular string are got ten by means of the dimensionless iterative interpolation algorithm. The basic data of the X Well, 1300 meters deep, are used for case history calculations. The results and some useful conclusions can provide technical reliability in the process of designing well testing in oil or gas wells.

## 1. Introduction

The deviated wells had been wildly applicable for petroleum and natural gas industry. Deviated wells have their distinctive characteristics which are distinguished from that of other wells. (1) High temperature high pressure: the temperature distribution and pressure on the tubing are significantly different when outputs are varied (flow velocity) but neither has a simple linear relationship, because the fluid density is not constant. (2) Deep well: the sensibility of force and deformation influencing by the factors, such as the temperature, pressure, density of fluid, viscous friction and fluid velocity, and so forth, will become high with the increase of tubing length. The completion test of a deep well is a new problem. In the research of applied basic theory for deep well testing, tubular string mechanical analysis is very complex, but fluid temperature and tubing pressure affect the force of the tubular string heavily. Temperature, pressure, liquid density, and fluid velocity within tubing may change with of the hole depth, time, and operations, so that the axial force changes constantly. A large compression load at low end can induce the tubing plastic deformation and make the packer damaged. A large tension load at the top end may unpack the packer or cause the tubing to break. If the tubing failed, the whole borehole can hardly maintain its integrity and safety [1]. Therefore, it is very important for deviated wells to predict the axial forces for the safety.

Hammerlindl [2] had made a great contribution about tubular mechanics. He had put forth the four effects between the packer forces and length change of tubing: temperature effect, ballooning effect, axial load effect, and the helical buckling effect. There is a large amount of papers to research the effect of buckling behavior. Therefore it is considered that inflexion is caused on its axial force under certain conditions, by which colliding on parts of the drill string with well bore is induced. When buckled of tubular beyond wellhole's control, the buckling configuration which will be transformed at the state of stabilization, sinusoidal buckling and helical buckling with the increase of load. The problem of buckling of the tube was first studied and put into practice by Lubinski et al. [3]. They had done the emulation experiment for the buckling behavior of tube in deviated wells and found the compute formula on critical buckling load of tube in deviated wells. Paslay and Bogy [4] found that the number of sinusoids in the buckling mode increases with the length of the tube. The buckling behavior by inner and outer fluid pressure of tubing was analyzed, and the mathematical relation between pitch and axial pressures was deduced based on the principle of minimum potential energy (see Hammerlindl [2]). The mptotic solution for sinusoidal buckling of an extremely long tube has been analyzed by Dawson and Paslay [5], based on a sinusoidal buckling mode of constant amplitude. Numerical solutions were also sought by Mitchell [6] using the basic mechanics equations. His solutions confirm the thought that, under a general loading, the deformed shape of the tube is a combination of helices and sinusoids while helical deformation occurs only under special values of the applied load. The formula about tubing forces had been put, however, which is too simple for shallow wells to accommodate the complicated states of deep wells. Up to now, many researches are centered on water injection tubular but not on steam injection. Among them, the values of temperature and pressure are considered as constant or lineal functions which will cause large errors on tubular deformation computing [7].

In fact, the tubular string deformation includes transverse deformation and longitudinal deformation. Because the transverse length (its order of magnitude is 10^−3^ m) is much and much smaller than the longitudinal length (its order of magnitude is 10^3^ m), we mainly consider the axial (longitudinal) deformation for the tubular string deformation analysis in the paper. In the paper, the force states of tubular in the process of steam injection are analyzed. The varied (*T*, *P*) fields are considered to compute the values of several deformations. The axial load and four deformation lengths of tubular string are obtained by the dimensionless iterative interpolation algorithm. The basic data of the X Well (deviated well), 1300 meters deep in China, are used for case history calculations. Some useful suggestions are drawn.

This paper is organized as follows.  Section 2 gives a system model about tubular mechanics and deformation. And the varied (*T*, *P*) fields were presented by model concerning mass, momentum, and energy balance.  Section 3 gives the parameters, initial condition, and algorithm for solving model. In Section 4, we give an example from a deviated well at 1300 meters of depth in China, and the result analysis are made.  Section 5 gives a conclusion. 

## 2. Model Building

### 2.1. Basic Assumption

Before analyzing the force on the microelement, some assumptions are introduced as follows:

1. the curvature of the hole of the considered modular section is constant,
2. on the upper side or underside of the section which is point of contact of the pipe and tube wall, the curvature is the same with the hole curvature,
3. the radius of steam injection string, in contrast to curvature of borehole, is insignificant,
4. the string is at the state of linear elastic relationship.

### 2.2. Forces Analysis of Tubular String

**Figure 1:**
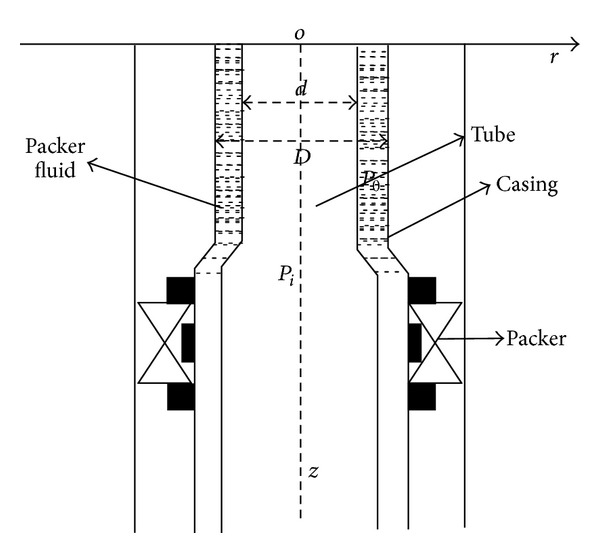
The physical figure of forces analysis on tube.

The forces of tubular string are shown in Figure 1. Consider the flow system depicted in Figure 1: a constant cross-sectional flow area *A*, inner diameter *d*, outer diameter *D*, material density *ρ*_1_, packer fluid density *ρ*_2_, and a total length *Z*. Through this tubing gas flows from the bottom to the top with a mass flow rate *W*. The distance coordinate in the flow direction along the tubing is denoted by *z*. The cylindrical coordinate system *rθz*, origin of which is in wellhead and *Z* axis is down as the borehole axis, is used. 

 As shown from Figure 1, the tubular string is mainly acted upon by the following forces at the process of steam injection.

1. *Initial Axial Force*. The initial axial force of tubular should include the deadweight, buoyant weight, and initial pull force.
2. *Thermal Stress*. On the process of steam injection, the temperature stress will act at the tubular with varied temperature.
3. *Axial Force by the Varied Internal and External Pressure*. Thanks to the varied pressure with internal and external pressure, the tubular will be acted by the bending force, piston force, and other axial forces.
4. *Friction Drag by Steam Injection*. On the process of steam injection, the flow in tubular will produce viscous flow which will cause the friction drag.

### 2.3. The Axial Load and Axial Stress of the Tubular

#### 2.3.1. Initial Axial Load and Initial Axial Stress of Steam Injection Tubular 

*Initial Axial Load*. The section to which the distance from the wellhead is *z*(*m*) was considered. The axial static load by the deadweight of tubular is as follows:

(1) $$\begin{array}{c} N_{qz}=\overset{L}{\underset{z}{\int }}q\cos\alpha \;dz=\frac{\pi }{4}\rho_{1}g\left(D^{2}-d^{2}\right)\overset{L}{\underset{z}{\int }}\cos\alpha \;dz, \end{array}$$

where *N*_*qz*_ is the deadweight of tubular, *q* is the average unit length weight of tubing, *L* is the length of tubular, *ρ*_1_ is the density of tubular, and *α* is the inclination angle.

The axial static load by the buoyant weight is as follows:

(2) $$\begin{array}{c} N_{bz}=-\rho_{2}gA_{2}\overset{L}{\underset{z}{\int }}\cos\alpha \;dz=-\rho_{2}gz\pi {\left(\frac{D}{2}\right)}^{2}\overset{L}{\underset{z}{\int }}\cos\alpha \;dz, \end{array}$$

where *N*_*bz*_ is the buoyant weight of tubular, *ρ*_2_ is the density of packer fluid.

The axial load by the steam injection pressure

(3) $$\begin{array}{c} N_{pz}=\frac{P_{z1}\pi d^{2}z}{4}, \end{array}$$

where, *P*_*z*1_ represents the inner pressure at this section.

Therefore, summing (1), (2), and (3), the axial forces in the section are obtained as follows:

(4) $$\begin{array}{c} F_{z}=N_{qz}+N_{bz}+N_{pz}. \end{array}$$

*Initial Axial Stress*. The axial stress can be derived from the following equation

(5) $$\begin{array}{c} \sigma_{zi}=\frac{4F_{z}}{\pi \left(D^{2}-d^{2}\right)}. \end{array}$$

#### 2.3.2. Axial Thermal Stress of Steam Injection Tubular

In the process of steam injection, the temperature of tubular will change with time and depth, which will make the tubular deform as follows:

(6) $$\begin{array}{c} \sigma_{zt}=E\beta \left(T_{z1}-T_{z0}\right)=E\beta \Delta T, \end{array}$$

where, *E* represents the steel elastic modulus of tubular, *β* is the warm balloon coefficient of the tubular string, and Δ*T* is the temperature change with before and after steam injection. 

#### 2.3.3. Axial Stress of Steam Injection Tubular by the Change with Pressure

The effect acting the tubular with pressure change which is called ballooning effect normally.

*Ballooning Stress Analysis*. The ballooning effect will be produced from pressure acted in inner and outer of the tube. Generally, there are two kinds of tubular in oil wells. One is the tubulars whose outer diameter is 88.9 mm, inner diameter is 76 mm, and thickness of tubes is 6.5 mm (*δ*/(*d*/2) = 17.1% > 5%); the other is the tubular whose outer diameter is 114.3 mm, inner diameter is 100.5 mm, and thickness of tubes is 6.9 mm (*δ*/(*d*/2) = 13.7% > 5%). Neither is the thin-wall problem. Therefore, it should be solved by Lame's formula [8]. 

**Figure 2:**
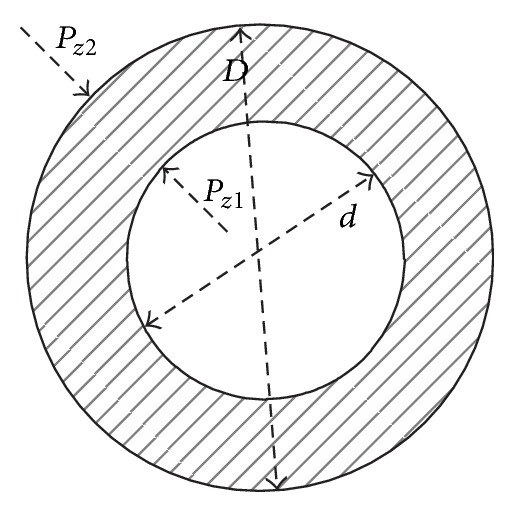
The radial and tangential stresses figure of tube.

The radial and tangential stresses in the thick-wall cylinder can be shown as Figure 2. The two can be calculated as follows:

(7) $$\begin{array}{c} \sigma_{rz}=\frac{d^{2}P_{z1}-D^{2}P_{z0}}{D^{2}-d^{2}}-\frac{\left(P_{z1}-P_{z0}\right)D^{2}d^{2}}{\left(D^{2}-d^{2}\right)4r^{2}},\\ \sigma_{\theta z}=\frac{d^{2}P_{z1}-D^{2}P_{z0}}{D^{2}-d^{2}}+\frac{\left(P_{z1}-P_{z0}\right)D^{2}d^{2}}{\left(D^{2}-d^{2}\right)4r^{2}}, \end{array}$$

where *r* is radial stress, *θ* is tangential stress, *r*  (*d* ≤ *r* ≤ *D*) is radial coordinate, *P*_*z*1_ is tube internal pressure at *z* point, and *P*_*z*0_ is tube external pressure at *z* point. 

#### 2.3.4. Axial Stress of Steam Injection Tubular by the Friction Loss

In fact, the flow in the tubular should be multiflow. On the process of steam injection, the flow will be run and it will give rise to friction effect to cause axial stress. In our paper, we consider the flow gas-liquid mix flow and the liquid head loss is gotten by the Darcy-Weisbach formula [9] as follows:

(8) $$\begin{array}{c} h_{f}=\frac{\lambda \left(Z-z\right)\nu_{m}^{2}}{2gd}, \end{array}$$

where *h*_*f*_ means heat loss of liquid flow, *λ* is frictional head losses coefficients, and *ν*_*m*_ is the velocity of liquid flow.

The friction drag in tubular is *N*_*fz*_ = *h*_*f*_ *ρ*_*m*_ *gπd* ^2^ (*ρ*_*m*_ is density of liquid flow). The axial stress by fiction drag can be obtained as follows:

(9) $$\begin{array}{c} \sigma_{zf}=\frac{4N_{fz}}{\pi \left(D^{2}-d^{2}\right)}. \end{array}$$

### 2.4. Analysis of Axial Deformation

Based on the studies and analyses mentioned above, the axial deformation on the tubular is made up of the following parts. 

#### 2.4.1. The Axial Deformation by the Axial Static Stress

For the microelement of the tubular *dz*, the unit deformation by the static stress can be computed by generalized Hooke law

(10) $$\begin{array}{c} {ɛ}_{1}=\frac{1}{E}\left[\sigma_{zi}-\mu \left(\sigma_{rz}+\sigma_{\theta z}\right)\right], \end{array}$$

where *μ* represents Poisson's ratios.

The axial deformation at an element can be obtained through integrating on the length of the element as follows:

(11) $$\begin{array}{c} \Delta L_{1i}=\overset{Z_{i}}{\underset{Z_{i-1}}{\int }}\frac{1}{E}\left[\sigma_{zi}-\mu \left(\sigma_{rz}+\sigma_{\theta z}\right)\right]dz. \end{array}$$

Therefore, the total axial deformation by the static stress can be gotten accumulating each element as follows:

(12) $$\begin{array}{c} \Delta L_{1}=\overset{N}{\underset{i=1}{\sum }}\Delta L_{1i}. \end{array}$$

#### 2.4.2. The Axial Deformation with Temperature Changed

For the microelement of the tubular *dz*, the unit deformation by the temperature change is as follows:

(13) $$\begin{array}{c} \Delta L_{2i}=\overset{Z_{i}}{\underset{Z_{i-1}}{\int }}\frac{\sigma_{zt}}{E}dz=\beta \Delta T_{i}\Delta L_{i}. \end{array}$$

The same principle is that the total axial deformation by the varied temperature fields can be gotten accumulating each element as follows:

(14) $$\begin{array}{c} \Delta L_{2}=\overset{N}{\underset{i=1}{\sum }}\Delta L_{2i}. \end{array}$$

#### 2.4.3. The Axial Deformation with the Friction Drag

For the microelement of the tubular *dz*, the unit deformation by the friction force is as follows:

(15) $$\begin{array}{c} \Delta L_{3}=\overset{Z}{\underset{0}{\int }}\frac{\sigma_{zf}}{E}dz=\frac{\lambda \rho_{m}\nu_{m}^{2}dZ^{2}}{E\left(D^{2}-d^{2}\right)}. \end{array}$$

#### 2.4.4. The Axial Deformation with the Tubular String Buckling

**Figure 3:**
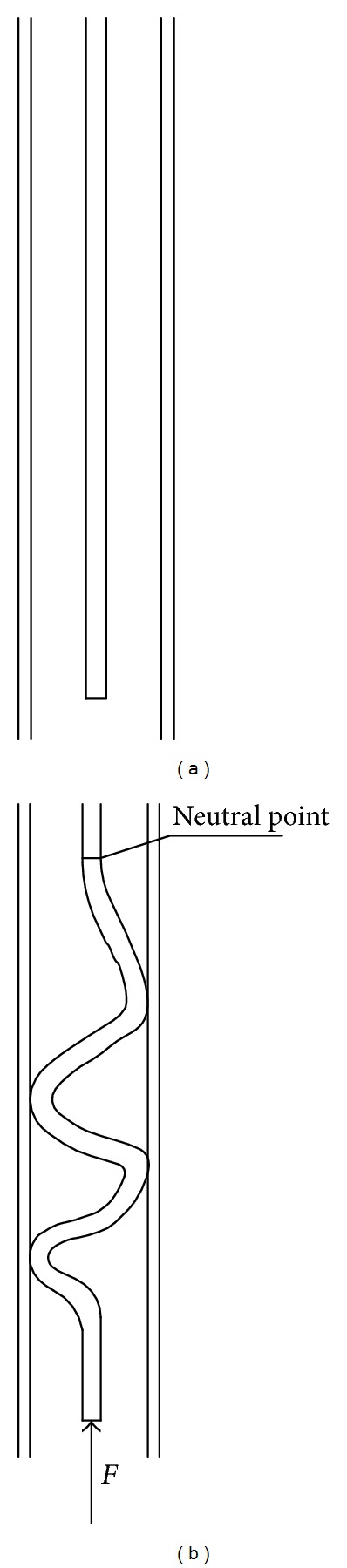
Buckling of tubular.

Researchers in general call the buckling a bending effect. The tubular is freely suspended in the absence of fluid inside as shown in Figure 3(a). Because the force *F* applied at the end of the tubular which is large enough, the tubular will buckle as shown in Figure 3(b).

Lubinski et al. [3] had done many researches on the phenomenon. From their work, we can get the buckling effect. Define the virtual axial force of tubing as follows:

(16) $$\begin{array}{c} F_{f}=A_{p}\left(P_{1}-P_{0}\right), \end{array}$$

where *P*_1_ is the pressure inside the tubular at the packer length, *P*_0_ is the pressure outside the tubular at the packer length, and *A*_*p*_ is the area corresponding to packer bore.

By (16), whether the tubular will buckle or not can be judged. The string will buckle if *F*_*f*_ is positive or remain straight if *F*_*f*_ is negative or zero. The axial deformation of the tubular string buckling is

(17) $$\begin{array}{c} \Delta L_{4i}=-\frac{r^{2}A_{p}^{2}{\left(\Delta P_{1i}-\Delta P_{0i}\right)}^{2}}{8EIW}, \end{array}$$

where *r* means tubing-to-casing radial clearance, *I* is moment of inertia of tubing cross-section with respect to its diameter (*I* = *π*(*D* ^4^ − *d* ^4^)/64), Δ denotes change with before and after injection, and *W* is the unit weight of tubing, as

(18) $$\begin{array}{c} \Delta L_{4}=\overset{N}{\underset{i=1}{\sum }}\Delta L_{4i}. \end{array}$$

In addition, the position of the neutral point is needed. The length (*n*) from the packer to the point can be computed as follows:

(19) $$\begin{array}{c} n=\frac{F_{f}}{W}. \end{array}$$

Generally, the neutral point should be in tubular (*n* ≤ *Z*). However, at the multipackers, it will occur that the neutral point is outside the tubing between dual packers. In this paper, we leave the latter phenomenon.

To sum up, the whole deformation length can be represented as follows:

(20) $$\begin{array}{c} \Delta L=\Delta L_{1}+\Delta L_{2}+\Delta L_{3}+\Delta L_{4}. \end{array}$$

### 2.5. The Analysis of the Varied (*T*, *P*) Fields

In the course of dryness modeling, we can find that the numerical values of deformation ((10), (13), and (17)) were affected by the temperature and pressure. In fact, the two parameters varied according to the depth and time changing. So, the varied (*T*, *P*) fields need to be researched. Under the China Sinopec Group Hi-Tech Project “Stress analysis and optimum design of well completion” in 2009 [6] undertaken by Sichuan University at early time. The varied (*T*, *P*) fields had been deduced strictly based on the mass, momentum, and energy balance. The proof details can be shown in Xu et al. [11]. The varied (*T*, *P*) fields is

(21) dPdz=−(τi/A)+ρmgcos⁡θ+(m/A)R(dx/dz)1−(m/A)S,dTdz=−νmCPg(Rdxdz−SdPdz)−gcos⁡θCPg−πfrtiρmνm34CPg+a(T−Te)CPg,P(z0)=P0, T(z0)=T0, dx(z0)=dx0, x(z0)=z0.

## 3. Numerical Implementation

### 3.1. Calculation of Some Parameters

In this section, we will give the calculating method of some parameters.

(1) Each point's inclination:
  (22) $$\begin{array}{c} \alpha_{j}=\alpha_{j-1}+\frac{\left(\alpha_{k}-\alpha_{k-1}\right)\Delta s_{j}}{\Delta s_{k}}, \end{array}$$
  where *j* represents segment point of calculation, Δ*s*_*k*_ represents measurement depth of inclination angle *α*_*k*_, and *α*_*k*−1_, Δ*s*_*j*_ is the step length of calculation. Transient heat transfer function [12]:
  (23) $$\begin{array}{c} f\left(t_{D}\right)=\{\begin{array}{cc} 1.128\sqrt{t_{D}}\left(1-0.3\sqrt{t_{D}}\right), & t_{D}\leq 1.5,\\ \left(0.4063+0.5\ln t_{D}\right)\left(1+\frac{0.6}{t_{D}}\right), & t_{D}>1.5. \end{array} \end{array}$$
(2) The density of wet steam. Since the flow of the water vapor in is the gas-liquid two-phase flow, there are many researches about this problem [13, 14]. In the paper, we adopt the M-B model to calculate the average density of the mixture.
(3) The heat transfer coefficient *U*_to_ from different positions of the axis of the wellbore to the second surface.

These resistances include the tubing wall, possible insulation around the tubing, annular space (possibly filled with a gas or liquid but is sometimes vacuum), casing wall, and cementing behind the casing as follows:

(24) $$\begin{array}{c} \frac{1}{U_{to}}=r_{ti}\frac{1}{\lambda_{\text{ins}}}\ln\left(\frac{r_{ci}}{r_{to}}\right)+\frac{1}{h_{c}+h_{r}}+r_{ti}\frac{1}{\lambda_{\text{cem}}}\ln\left(\frac{r_{\text{cem}}}{r_{co}}\right) \end{array}$$

*λ*_ins_ and *λ*_cem_ are the heat conductivity of the heat insulating material and the cement sheath, respectively. *h*_*c*_ and *h*_*r*_ are the coefficients of the convection heat transfer and the radiation heat transfer. 

### 3.2. Initial Condition

In order to solve model, some definite conditions and initial conditions should be added. The initial conditions comprise the distribution of the pressure and temperature at the well top. In this paper, we adopt the value at the initial time by actual measurement. Before steam injected, the temperature of tubular just is initial temperature of formation (*T*_*z*_ = *T*_0_ + *γz*cos⁡*α*, *γ* is geothermal gradient). At the same time, the pressure of inner tubular is assumed to be equal to the outer tubular before steam injected. 

### 3.3. Steps of Algorithm

To simplify the calculation, we divided the wells into several short segments of the same length. The length of a segment varies depending on variations in wall thickness, hole diameter, fluid density inside and outside the pipe, and wells geometry. The model begins with the calculation at one particular position in the wells: the top of the pipe.

**Step 1**

Set step length of depth. In addition, we denote the relatively tolerant error by *ɛ*. The smaller *h*, *ɛ* is, the more accurate the results are. However, it will lead to rapid increasing calculating time. In our paper, we set *h* = 1 (m), and *ɛ* = 5%.

**Step 2**

Give the initial conditions.

**Step 3**

Compute each point's inclination.

**Step 4**

Compute the parameters under the initial conditions or the last depth variables.

**Step 5**

Let *T* = *T*_*k*_; then we can get the *T*_*e*_ by solving the following equation:

(25) $$\begin{array}{c} \frac{\partial T_{e}}{\partial t_{D}}=\left(\frac{\partial^{2}T_{e}}{\partial r_{D}^{2}}+\frac{1}{r_{D}}\frac{\partial T_{e}}{\partial r_{D}}\right),\\ {T_{e}|}_{t_{D}=0}=T_{0}+\gamma z\cos\theta ,\\ {\frac{\partial T_{e}}{\partial r_{D}}|}_{r_{D}=1}=-\frac{1}{2\pi \lambda_{f}}\frac{dq}{dz},\\ {\frac{\partial T_{e}}{\partial r_{D}}|}_{r_{D}\to \infty }=0. \end{array}$$

Let *T*_*e*,*i*_^*j*^ be the temperature at the injection time *j* and radial *i* at the depth *z*. We apply the finite different method to discretize the equations as follows:

(26) $$\begin{array}{c} \frac{T_{e,j}^{i+1}-T_{e,j}^{i}}{\varphi }=\frac{T_{e,j+1}^{i+1}-2T_{e,j+1}^{j}+T_{e,j+1}^{i-1}}{\xi^{2}}-\frac{T_{e,j+1}^{i+1}-T_{e,j}^{i+1}}{r_{D}\varphi }, \end{array}$$

where *φ* is the interval of time and *ξ* is the interval of radial, respectively. It can be transformed into the standard form as follows:

(27) $$\begin{array}{c} -\left(\varphi +\frac{\varphi \xi }{r_{D}}\right)T_{e,j+1}^{i+1}+\left(2\varphi +\frac{\varphi \xi }{r_{D}}\right)T_{e,j}^{i+1}-\varphi T_{e,j-1}^{i+1}=\xi^{2}T_{e,j}^{i}. \end{array}$$

Then the different method is used to discretize the boundary condition. For *r*_*D*_ = 1, we have

(28) $$\begin{array}{c} T_{e,2}^{e,i+1}-\left(1+\frac{a\xi }{2\pi \lambda_{f}}\right)T_{e,1}^{i+1}=\frac{aT_{k}}{2\pi \lambda_{f}}. \end{array}$$

For *r*_*D*_ = *N*, we have

(29) $$\begin{array}{c} T_{e,n}^{i+1}-T_{e,n-1}^{i+1}=0. \end{array}$$

We can compute the symbolic solution of the temperature *T*_*e*_ of the stratum. In this step, we will get the discrete distribution of *T*_*e*_ as the following matrix:

(30) $$\begin{array}{c} \left[\begin{array}{ccccc} T_{e,1}^{1} & T_{e,1}^{2} & \cdots & {\text{T}}_{e,1}^{i} & \cdots \\ T_{e,2}^{1} & T_{e,2}^{2} & \cdots & T_{e,2}^{i} & \cdots \\ \vdots & \vdots & \cdots & \vdots & \vdots \\ T_{e,j}^{1} & T_{e,j}^{2} & \cdots & T_{e,j}^{i} & \cdots \\ \vdots & \vdots & \cdots & \vdots & \vdots \\ T_{e,n}^{1} & T_{e,n}^{2} & \cdots & T_{e,n}^{i} & \cdots \end{array}\right], \end{array}$$

where *i* represents the injection time and *j* represents the radial.

**Step 6**

Let the right parts of the coupled differential equations be functions *F*_*k*_, where (*k* = 1,2). Then we can obtain a system of coupled functions as follows:

(31) F1=−(τi/A)+ρmgcos⁡θ+(m/A)R(dx/dz)1−(m/A)S,F2=−νmCPg(Rdxdz−SdPdz)−gcos⁡θCPg−πfrtiρmνm34CPg+a(T−Te)CPg,

where *T*_*e*_ at *r*_*D*_ = 1.

**Step 7**

Assume that *P*, *T* are *y*_*k*_  (*k* = 1; 2), respectively. Then we can obtain some basic parameters as follows:

(32) $$\begin{array}{c} a_{k}=F_{i}\left(y_{1},y_{2}\right),\\ b_{k}=F_{i}\left(y_{1}+\frac{ha_{1}}{2},y_{2}+\frac{ha_{2}}{2}\right),\\ c_{k}=F_{i}\left(y_{1}+\frac{hb_{1}}{2},y_{2}+\frac{hb_{2}}{2}\right),\\ d_{k}=F_{i}\left(y_{1}+hc_{1},y_{2}+hc_{2}\right). \end{array}$$

**Step 8**

Calculate the pressure and temperature at point (*j* + 1):

(33) ykj+1=ykj+h(ak+2bk+2ck+dk)6,k=1,2,  j=1,2,…,n.

**Step 9**

Calculate the deformation Δ*L*_1*j*_, Δ*L*_2*j*_, and Δ*L*_4*j*_ by previous equations.

**Step 10**

Repeat the third step to the tenth step until tubular length *Z* is calculated.

**Step 11**

Calculate the deformation Δ*L*_3_ and total deformation length as follows:

(34) $$\begin{array}{c} \Delta L=\overset{N}{\underset{j=1}{\sum }}\Delta L_{1j}+\overset{N}{\underset{j=1}{\sum }}\Delta L_{2j}+\Delta L_{3}+\overset{N}{\underset{j=1}{\sum }}\Delta L_{4j}. \end{array}$$

## 4. Numerical Simulation

### 4.1. Parameters

To demonstrate the application of our theory, we study a pipe in X well, which is in Sichuan Province, China. All the basic parameters are given as follows: depth of the well is 1300 m; ground thermal conductivity parameter is 2.06; ground temperature is 16°C; ground temperature gradient is 0.0218 (°C/m); roughness of the inner surface of the well is 0.000015; and parameters of pipes, inclined well, inclination, azimuth, and vertical depth are given in Tables 1, 2, and 3. 

### 4.2. Main Results and Results Analysis

**Figure 4:**
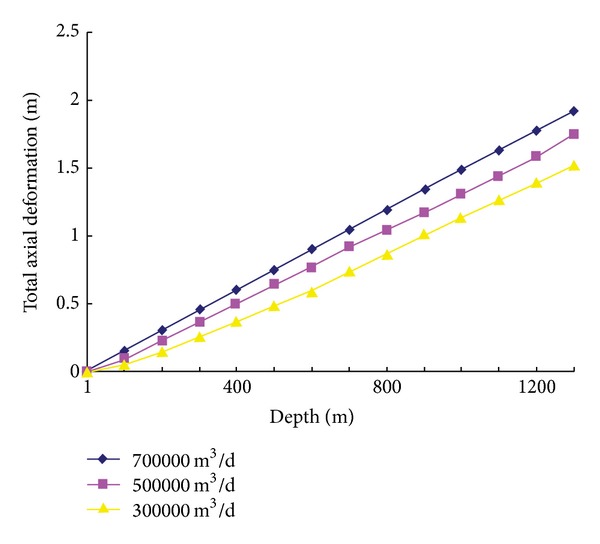
The total axial deformation under varied outputs.

After calculation, we obtain a series of results of this well as Table 4. The influence of outputs on the axial deformation of tubing was investigated as shown by Figure 4.

From the results as shown in Figure 4 and Table 4, some useful analysis can be drawn.

1. The amount of steam injected and inject pressure affected the stretching force with special severity.
2. The results were as follows: the length of tubular deformation was risen with increased injected pressure or injected velocity.
3. The length of tubular deformation increases with the increasing of outputs but more slowly.
4. The thermal stress is the main factor influencing the tubular deformation. Therefore, the temperature of steam injected should not be too high.
5. The lifting prestressed cementing technology has important meanings to reduce the deformation of tubular.
6. The creeping displacement of downhole stings will produce an upward contractility which causes packer depressed or lapsed. Therefore, the effective measures should be adopted to control the companding of tubular.

## 5. Conclusion

In this paper, a total tubular deformation model about deviated wells was given. A coupled-system model of differential equations concerning pressure and temperature in high temperature-high pressure steam injection wells according to mass, momentum, and energy balances, which can reduce the error of axial stress and axial deformation, was given instead of the average value or simple linear relationship in traditional research. The basic data of the Well (high temperature and high pressure gas well), 1300 m deep in Sichuan, China, were used for case history calculations. The results can provide technical reliance for the process of designing well tests in deviated gas wells and dynamic analysis of production. 

## Figures and Tables

**Table 1:** Parameters of pipes.

Diameter (m)	Thickness (m)	Weight (Kg)	Expansion	Elastic (Gpa)	Poisson's ratios	Using length (m)
0.0889	0.01295	23.79	0.0000115	215	0.3	270
0.0889	0.00953	18.28	0.0000115	215	0.3	120
0.0889	0.00734	15.04	0.0000115	215	0.3	620
0.0889	0.00645	13.58	0.0000115	215	0.3	290

**Table 2:** Well parameters.

Measured (m)	Internal (m)	External (m)
336.7	0.15478	0.1778
422.6	0.1525	0.1778
1300.0	0.10862	0.127

**Table 3:** Parameters of azimuth, inclination, and vertical depth.

Number	Measured (m)	Inclination (°)	Azimuth (°)	Vertical depth (m)
1	135	2.63	241.01	134.72
2	278	1.23	237.86	277.91
3	364	1.43	213.86	363.82
4	393	2.17	26.38	392.53
5	422	1.85	44.56	421.28
6	450	0.82	191.12	449.62
7	486	2.93	269.07	485.47
8	514	1.03	297.55	513.83
9	543	3.58	324.51	541.74
10	571	2.98	303.05	570.43
11	600	2.03	204.74	599.42
12	628	2.34	164.33	627.28
13	660	1.85	195.28	659.56
14	723	3.14	214.84	721.70
15	782	0.98	216.48	781.30
16	830	2.15	229.31	829.12
17	860	2.67	244.03	859.71
18	908	4.85	266.62	904.08
19	928	6.72	258.78	921.42
20	972	2.03	236.88	971.71
21	1025	4.78	239.27	1021.25
22	1058	4.01	244.59	1055.58
23	1089	4.98	228.2	1084.17
24	1132	3.75	233.88	1129.28
25	1174	5.63	235.14	1168.87
26	1204	4.23	234.38	1200.99
27	1235	3.87	234.99	1232.08
28	1268	4.97	232.57	1263.45
29	1300	8.84	233.28	1284.96

**Table 4:** The results of the axial force and various kinds of deformation lengths.

Number	Depth (m)	Axial force (N)	Displacement by temperature changed (m)	Displacement by pressure changed (m)	Axial deformation (m)	Buckling deformation (m)	Total deformation (m)
1	1	895244.8	0	0	0	0	0
2	100	854724.8	0.1201	0.00986	0.024	0	0.1544
3	200	814215.5	0.2362	0.019392	0.052	0	0.3072
4	300	773717.7	0.3483	0.028598	0.082	0	0.459
5	400	737970	0.4564	0.037476	0.115	−0.006	0.6029
6	500	706877.3	0.5606	0.046028	0.152	−0.006	0.7523
7	600	675763.9	0.6607	0.054254	0.192	−0.006	0.9006
8	700	644602.3	0.7569	0.062153	0.235	−0.006	1.048
9	800	613437.2	0.849	0.069725	0.283	−0.007	1.1946
10	900	582272.1	0.9371	0.076968	0.335	−0.007	1.3422
11	1000	551107.2	1.0212	0.083883	0.391	−0.007	1.4896
12	1100	519942.3	1.1014	0.090471	0.452	−0.007	1.6367
13	1200	488777.5	1.1775	0.096731	0.517	−0.009	1.7822
14	1300	457612.8	1.2496	0.102662	0.584	−0.01	1.9261

## Nomenclature

*d*: Inner diameter (m)
*d*_*z*_: Microelement of the tubular
*g*: Acceleration of gravity (m/s^2^)
*h*: Depth of top tubular located at the packer (m)
*t*: Time of down stroke (s)
*t*_*D*_: Dimensionless time (*dimensionless*)
*v*_*i*_: Velocity of fluid in tubing (m/s)
*v*_*s*_: Velocity of down stroke (m/s)
*z*: Distance coordinate in the flow direction (m) along the tubing
*A*: Constant cross-sectional flow area (m^2^)
*A*_*c*_: Effective area (m^2^)
*A*_*p*_: Area corresponding to packer bore (m^2^)
*C*_*J*_: The Joule-Thomson coefficient (*dimensionless*)
*C*_*P*_: Heat capacity of fluids (J/Kg · K)
*D*: Outer diameter (m)
*E*: Steel elastic modulus of tubular (Mpa)
*F*_*i*_: Axial forces in the section (N)
*F*_*z*_: Axial tensile strength (N)
*F*_*f*_: Friction force (N)
*F*_*c*_: Piston force for supporting packer's pressure (N)
*F*_*s*_: Pumping force (N)
*L*: Length of tubular (m)
*N*_*bz*_: Buoyant weight of tubular (Kg)
*N*_*qz*_: Dead weight of tubular (Kg)
*P*_0_: Pressure outside the tubular (Mpa)
*P*_1_: Pressure inside the tubular at the packer length (Mpa)
*P*_*i*_: Pressure in tubing (Mpa)
*T*_*i*_: Temperature in tubing (°C)
*T*_*e*_: Initial temperature of formation (°C)
*W*: Mass flow rate (Kg/s)
*Z*: Total length (m)
*ρ*_1_: Material density (Kg/m^3^)
*ρ*_2_: Packer fluid density (Kg/m^3^)
*α*: Inclination angle (∘)
*β*: Warm balloon coefficient of the tubular string (*dimensionless*)
*δ*: Drop of any parameter
*σ*_*zt*_: Axial thermal stress (*N*)
*ρ*_*i*_: Density of fluid in the tubing (Kg/m^3^)
Δ*L*_3_: The tubular string buckling axial deformation (m)
Δ*L*_*t*_: Total axial deformation by varied temperature fields (m)
Δ*L*_*b*_: Total axial deformation by the varied pressure fields (m)
Δ*P*_start_: Differential pressure at startup (Mpa)
Δ*P*_*ii*_: Change in tubing pressure at the *i* length (Mpa)
Δ*P*_*oi*_: Change in annulus pressure at the *i* length (Mpa)
Δ*P*_*c*_: Differential pressure from top to bottom (Mpa)
Δ*T*: Temperature change with before and after well shut-in (°C)
Δ*ρ*_*ii*_: Change in density of liquid in the tubing at the *i* length (Kg/m^3^)
Δ*ρ*_*oi*_: Change in density of liquid in the casing at the *i* length (Kg/m^3^).
